# Anagrelide Modulates Proplatelet Formation Resulting in Decreased Number and Increased Size of Platelets

**DOI:** 10.1097/HS9.0000000000000268

**Published:** 2019-06-18

**Authors:** Naohiro Miyashita, Masahiro Onozawa, Shota Yokoyama, Daisuke Hidaka, Koji Hayasaka, Shinji Kunishima, Takanori Teshima

**Affiliations:** 1 Department of Hematology, Hokkaido University Faculty of Medicine, Graduate School of Medicine, Sapporo, Japan; 2 Division of Laboratory and Transfusion Medicine, Hokkaido University Hospital, Sapporo, Japan; 3 Department of Medical Technology, Gifu University of Medical Science, Seki, Japan.

## Abstract

We retrospectively evaluated 48 essential thrombocythemia (ET) patients who were treated in our institute (male/female, 14/34, median age, 61.5 years). In 14 patients treated with anagrelide (ANA), the degree of platelet count reduction (median, −56.6%) was strongly correlated with increase of mean platelet volume (MPV) (median, +11.7%) (R = 0.777). This correlation was not observed in ET patients treated with hydroxycarbamide alone (R = 0.245). The change in size of platelets strongly suggested that ANA affected the final process of platelet production. Thus, we hypothesized that ANA modifies the process by which platelets are released from proplatelets. To verify the association in an in vitro setting, we compared MEG-01 cells treated with PMA ± ANA. The number of platelet-like particles (PLPs) was decreased (*P* < 0.05) and the size of PLPs estimated by using flow cytometry was significantly increased when MEG-01 cells were treated with PMA + ANA (*P* < 0.05 vs PMA alone), recapitulating the clinical findings. The cytoplasmic protrusions extending from MEG-01 cells were shorter and thicker and the number of proplatelets was decreased when MEG-01 cells were treated with PMA + ANA (*P* < 0.01 vs PMA alone). Western blotting analysis showed that ANA treatment resulted in increased phosphorylation of MLC2 and reduced phosphorylation of focal adhesion kinase (FAK). The morphological change of proplatelets were reversed by blebbistatin, a specific inhibitor of myosin II. These findings indicated that ANA modulates the FAK-RhoA-ROCK-MLC2-myosine IIA pathway and suppresses proplatelet maturation, leading to a decrease in platelet count and increase in MPV.

## Abstract 2

Supplemental Digital Content is available in the text

## Introduction

Essential thrombocythemia (ET) is a chronic myeloproliferative neoplasm (MPN) characterized by an increased number of platelets in the blood. ET is a clonal stem cell disorder mainly induced by gene mutations such as *JAK2*, *MPL*, and *CALR* mutations. Major complications due to clonal expansion of platelets include thrombosis and bleeding. In the later stages, ET can transform into myelofibrosis or acute leukemia. The main goal of treatment for patients with ET is prevention of thrombotic events. Age over 60 years, a previous thrombotic event, existence of JAK2 mutation, cardiovascular risk factors and platelet count over 1500 × 10^9^/L have been reported to be risk factors for thrombosis in patients with ET.^1–3^ In ET patients with high risk factors of thrombotic events, administration of an antiplatelet agent such as aspirin and cytoreductive therapy with hydroxycarbamide (HC), interferons (IFNs) or anagrelide (ANA) are treatment options.^4,5^ The ELN guideline recommends HC or IFNα as first-line treatment and recommend the use of ANA in high-risk patients who are intolerant or resistant to HC treatment.^4^ The NCCN guideline recommends HC, IFNα or ANA as first-line therapy for high-risk ET patients. In Japan, both ANA and HC are recommended as first-line therapy for high-risk ET patients based on the results of comparative studies of HC and ANA.^6–8^ Because of genotoxicity, the potential risk for secondary leukemia remains a major concern of HC treatment, especially for young patients. ANA was initially developed as an antiplatelet agent,^9^ and its thrombocytopenic effect was discovered during preclinical trials.^10^ Initially, inhibition of megakaryocyte maturation was focused on as the mechanism underlying the platelet-lowering effect of ANA.^11,12^ In megakaryocytes derived from CD34-positive cells, ANA decreased mRNA levels of transcription factors such as *GATA-1*, *FOG1*, *FLI-1*, and *NF-E2*, which are associated with megakaryocyte maturation.^13,14^ At the late phase of megakaryocytic differentiation, megakaryocytes form long, branched cytoplasmic extensions named proplatelets, which protrude into the bone marrow sinusoidal lumen where platelets are released from proplatelet tips.^15^ Recently, ANA-induced phosphorylation of myosin light chain 2 (MLC2) was detected in megakaryocytes differentiated from CD34+ cord blood, and the phosphorylation leads to the formation of wider proplatelet shafts, which possibly contribute to the platelet-lowering effect of ANA.^16^ Modulation of proplatelets might be a direct mechanism underlying platelet reduction by ANA. Proplatelet maturation is known to be controlled by the focal adhesion kinase (FAK)-RhoA-ROCK-MLC2 pathway; however, the upstream region of the MLC2 pathway has not been explored.

In this report, we report interesting finding that platelet-lowering effect of ANA associated with increased platelet size in ET patients. Due to morphological change of platelets, we hypothesized that ANA affect formation of proplatelet, the final process of platelet release.

## Patients and Methods

### Patients

We retrospectively evaluated 48 patients who were diagnosed with ET in Hokkaido University Hospital during the period from 2010 to 2018 (male/female: 14/34, ages: 18 to 87 years, median age: 61.5 years). Medical records of the patients were reviewed and we checked age, sex, *JAK2* V617F mutation status, platelet count at the start of administration of ANA or HC, platelet count at the time of maximum response to ANA or HC, MPV at the start of administration of ANA or HC, MPV at the time of maximum response to ANA or HC, and time to maximum response. Three patients were treated with ANA alone. Eleven patients were administered ANA due to an insufficient decrease in platelets after treatment with HC or intolerance to HC. Eighteen patients were treated solely with HC. Sixteen patients were observed without ANA or HC treatment. We analyzed the changes in platelet count and MPV after administration of each medicine. All the patients treated with ANA had at least one of the risk factors of thrombosis including *JAK2* mutation, age over 60 years, history of thrombosis, high platelet count (≥1500 × 10^9^/L), and cardiovascular risk factors.^1–3^ Eleven of the 14 ET patients treated with ANA were over 60 years of age. Three of the 14 ET patients treated with ANA had a history of thrombosis. Platelet count was over 1500 × 10^9^/L in 5 of the 14 ET patients treated with ANA (Table S1, Supplemental Digital Content 1). *JAK2* V617F mutation status was checked in 12 patients and *JAK2* V617F mutation was positive in 8 patients (66.7%, Fig. S1A, Supplemental Digital Content 2). ANA was administered in 7 of the 8 ET patients with *JAK2* V617F mutation in this study. WBC count was significantly higher in *JAK2* V617F mutation-positive patients than in *JAK2* V617F mutation-negative patients in this study (Fig. S1B, Supplemental Digital Content 2). This study was approved by the Institutional Review Board of Hokkaido University Hospital.

### Methods

#### Cell line and megakaryocytic differentiation

MEG-01 is a human megakaryocytic leukemia cell line and is widely used as an experimental model to study the process of platelet production from megakaryocytes.^17–19^ MEG-01 cells can differentiate into megakaryocytes by stimulation with phorbol-12-myristate-13-acetate (PMA), recombinant human thrombopoietin, or low-concentrated fetal bovine serum.^17–19^ MEG-01 cells extend cytoplasmic protrusions similar to those of megakaryocyte proplatelets and release platelet-like particles (PLPs), which express platelet-specific glycoproteins such as CD61 (β3) and CD41 (αIIb).^17–19^ In this study, MEG-01 cells were cultured in Roswell Park Memorial Institute 1640 medium with 10% fetal bovine serum and 1% penicillin/streptomycin with or without 10 nM PMA (Wako, Osaka, Japan). After 2-day incubation at 37°C with 5% CO_2_, the cells were retrieved and lysed with SDS lysis buffer containing 1.7% SDS, 60 mM Tris-HCl, pH 6.8, 0.85% 2-mercaptoethanol, and proteinase inhibitor cocktail (Roche, Basel, Switzerland). To examine the expression levels of integrin αIIb and integrin β3, which are megakaryocytic markers, Western blotting was performed using an anti-integrin αIIb antibody (B-9, Santa Cruz Biotechnology, Santa Cruz, CA) and an anti-integrin β3 antibody (AB2984, Millipore, Bedford, MA), respectively. Protein loading of each well was verified by an anti-β-actin antibody (AC-15; Sigma-Aldrich, St. Louis, MO).

#### Proplatelet formation and platelet-like particles

MEG-01 cells were seeded in 35 mm glass-based dishes (IWAKI, Chiba, Japan) at a density of 2.5 × 10^4^ cells/ml and cultured with 10 nM PMA alone, 10 nM PMA with 100 μM HC, 10 nM PMA with 5 μM ANA (SIGMA-ALDRICH, St. Louis, MO), or 10 nM PMA with 5 μM ANA and 20 μM blebbistatin (FUJIFILM Wako Pure Chemical, Osaka, Japan) for 2 days. MEG-01 cells were incubated with 20 μM blebbistatin for 30 minutes followed by treatment with 5 μM ANA. To evaluate morphological changes, the cells were stained with May-Grünwald stain followed by Giemsa stain on day 2. Then the number, length and maximum shaft thickness of protrusions extending from MEG-01 cells were determined as previously reported.^20^ One hundred MEG-01 cells were analyzed in each sample.

To determine the number of PLPs, MEG-01 cells were seeded in 35 mm glass-based dishes at a density of 2.5 × 10^4^ cells/ml and cultured with 10 nM PMA alone or 10 nM PMA with 5 μM ANA for 2 days. The cells were fixed with 3% paraformaldehyde and permeabilized with 0.1% Triton-X-100. Then the cells were stained with AB2984 and Hoechst 33342 (Dojindo Laboratories, Kumamoto, Japan) followed by Alexa 594-conjugated secondary antibody (Thermo Fisher Scientific, Waltham, MA) staining on day 2. The number of PLPs per high-power field (HPF × 200) was counted. Images were obtained using a Keyence BZ-X700 all-in-one fluorescence microscope (Keyence, Osaka, Japan).

To determine the size of PLPs, PLPs were stained with an anti-human CD41 (αIIb) antibody (5B12; Dako, Glostrup, Denmark) and detected using flow cytometry (FCM). PLP is a low forward scatter (FS) particle expressing CD41 and we considered a CD41-positive particle in the gate for the peripheral blood platelet as a PLP in this study. Mean fluorescent intensity (MFI) of FS was measured to determine the size of PLPs.

#### Expression of FAK, pFAK, and pMLC2

MEG-01 cells treated with 10 nM PMA alone or 10 nM PMA with 1 μM or 5 μM ANA for 2 days were peeled off, and suspended cells were collected after incubation for 1 hour at 37°C with 5% CO_2_. The suspended cells were lysed with SDS lysis buffer containing 1.7% SDS, 60 mM Tris-HCl, pH 6.8, 0.85% 2-mercaptoethanol, and proteinase inhibitor cocktail to examine the expression of FAK, tyrosine-phosphorylated FAK (pFAK), and phosphorylated MLC2 (pMLC2) by Western blotting. FAK, pFAK, and pMLC2 were detected with an anti-FAK antibody (EP695Y, Abcam, Cambridge, UK), anti-phospho-FAK (Tyr397) antibody (3283, Cell Signaling, Danvers, MA), and anti-phospho-MLC2 antibody (3675S, Cell Signaling, Danvers, MA), respectively. Protein loading of each well was assessed by an anti-β-actin antibody. The phosphorylation state of FAK was quantified as the density ratio of protein bands pFAK/FAK, and the phosphorylation state of MLC2 was quantified as the density ratio of protein bands pMLC2/β-actin.

#### Statistical analysis

The association between rate of change in MPV and rate of change in platelet count after administration of ANA was evaluated by Pearson product moment correlation coefficient. The percentages of patients treated with ANA were compared by Fisher exact test. White blood cell (WBC) count and platelet count were compared by the *t* test. Total expression levels of αIIb, β3, pFAK, and pMLC2 in MEG-01 cells treated with PMA alone and those in MEG-01 cells treated with PMA and ANA were compared by the *t* test. The number of protrusions, length of protrusions, maximum shaft thickness of protrusions, number of PLPs, and FS intensity of PLPs in MEG-01 cells treated with PMA alone and those in MEG-01 cells treated with PMA and ANA were also compared by the *t* test. p values < 0.05 were considered to be statistically significant. All statistical analyses were performed with EZR.^21^

## Results

**Figure 1:**
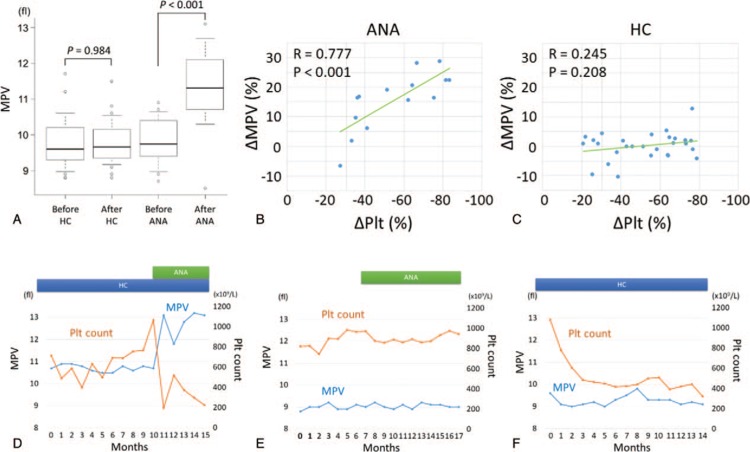
(A) Box plot shows the change of MPV after treatment with HC or ANA. Treatment with ANA increased MPV although treatment with HC did not affect MPV. (B) Scatter plot shows the rate of decrease (%) in platelet count and the rate of change (%) in MPV at the time of maximum response to ANA. The data were obtained from 14 patients who underwent ANA treatment. Pearson product moment correlation coefficient = 0.777, *P* < 0.001. (C) Scatter plot shows the rate of decrease (%) in blood platelet count and the rate of change (%) in MPV at the time of maximum response to HC. The data were obtained from 28 patients who underwent HC treatment only without ANA. Pearson product moment correlation coefficient = 0.245, *P* = 0.208. (D) Clinical course of a representative case that showed a clinical response to ANA. Platelet count (orange line) and MPV (blue line) are shown. (E) Clinical course of a representative case that did not show a clinical response to ANA. Platelet count (orange line) and MPV (blue line) are shown. (F) Clinical course of a representative case that showed a clinical response to HC. Platelet count (orange line) and MPV (blue line) are shown. ANA = anagrelide; HC = hydroxycarbamide; MPV = mean platelet volume; Plt = platelet.

The characteristics of patients treated with ANA are shown in Table S1 (Supplemental Digital Content 1). In 11 of the 29 patients treated with HC, treatment with ANA was initiated due to a lack of efficacy of HC (n = 3, 27.3%) or intolerance to HC (n = 8, 72.7%). Intolerance to HC was indicated by leg ulcers (n = 4), skin eruption (n = 1) and/or progressive anemia (n = 4). Treatment with ANA alone was initiated in 3 patients. In the 14 patients treated with ANA, platelet counts at the initiation of ANA treatment were 424 to 1810 × 10^9^/L (median, 960 × 10^9^/L). A reduction in platelet count of more than 30% was achieved in 13 patients (92.9%) after ANA treatment. In ET patients treated with ANA, the mean of MPV was significantly higher at the maximum response to ANA than at the induction of ANA (Fig. 1A). In ET patients treated with HC, there was no difference between the mean MPV at the maximum response to HC and the mean MPV at the induction of HC. Then, we analyzed association between change of MPV and the degree of platelet count reduction in each patient (Fig. 1B, C). The plots in Figure 1B, C show the data obtained at the time of maximum response to ANA or HC. Interestingly, the degree of platelet count reduction (−83.4% to −27.1%; median, −56.6%) was strongly correlated with increase of MPV (−6.6% to +28.7%; median, +16.7%) (R = 0.777, *P* = 0.001, Fig. 1B). In the 29 patients treated with HC, platelet counts at initiation of HC treatment were 211 to 1809 × 10^9^/L (median, 824 × 10^9^/L). A reduction in platelet count of more than 30% was achieved in 23 patients (79.3%) after HC treatment. The degree of platelet count reduction (−78.7% to −20.8%; median, −55.5%) was not correlated with change of MPV (−10.3% to +12.9%; median, +1.0%) (R = 0.245, *P* = 0.21, Fig. 1C). Time to maximum response was shorter in patients treated with ANA (0.5 to 29.4 months; median, 3.7 months, *P* < 0.01 vs HC) than in those treated with HC (1.9 to 102 months; median, 12.0 months). A peripheral blood smear at the time of maximum response to ANA and the clinical course of a representative case responding to ANA are shown in Figure S1D (Supplemental Digital Content 2) and Figure 1D, respectively. MPV increased in accordance with the reduction of blood platelet count, and giant platelets were observed in the peripheral blood smear. The clinical courses of a case refractory to ANA and a case responding to HC are shown in Figure 1E and F, respectively. In the case of ANA failure, MPV remained unchanged (Fig. 1E). In contrast, HC treatment did not change MPV despite a reduction in platelet count (Fig. 1F).

**Figure 2:**
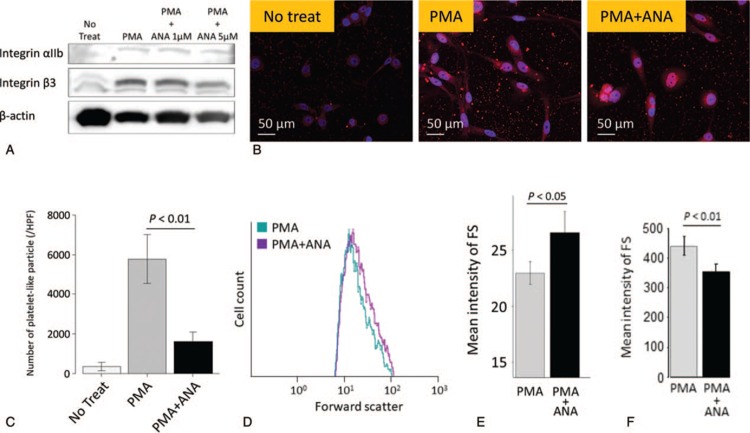
(A) Expression of integrin αIIb and integrin β3 in MEG-01 cells. PMA increased the expression of integrin αIIb and integrin β3. Results are representative of 3 independent experiments. (B) Immunofluorescence staining of MEG-01 cells and platelet-like particles (PLPs). PMA promote production of PLPs (center). The number of PLPs was decreased in the PMA + ANA treated MEG-01 cells (right). Red: integrin β3, Blue: Hoechst 33342. Left: Non-treated MEG-01 cells, Center: 10 nM PMA-treated MEG-01 cells, Right: 10 nM PMA and 5 μM ANA-treated MEG-01 cells. (C) The number of platelet-like particles stained with anti-CD61 (β3) antibody per high-power field (HPF, x200) was counted. PMA increase the number of PLPs. The number of PLPs was decreased in the PMA + ANA treated MEG-01 cells. Results are means plus or minus SD from 3 independent experiments. (D) Evaluation of the size of PLPs produced from MEG-01 cells. PLPs are low forward scatter (FS) particles expressing CD41 (αIIb) and are detected by using flow cytometry. With the addition of ANA, the graph shifted to the right, which meant increased size of PLPs. (E) Mean FS intensity of PLPs was measured to estimate the size of PLPs. FS intensity of platelet-like particles was significantly higher in the PMA + ANA treated MEG-01 cells compared to the cells treated with PMA alone. Results are means plus or minus SD from 3 independent experiments. (F) Mean FS intensity of MEG-01 cells was measured to estimate the size of MEG-01 cells. FS intensity of MEG-01 cells was significantly lower in the PMA + ANA treated MEG-01 cells compared to the cells treated with PMA alone. Results are means plus or minus SD from 3 independent experiments. ANA = anagrelide; FS = forward scatter; PMA = phorbol-12-myristate-13-acetate.

**Figure 3:**
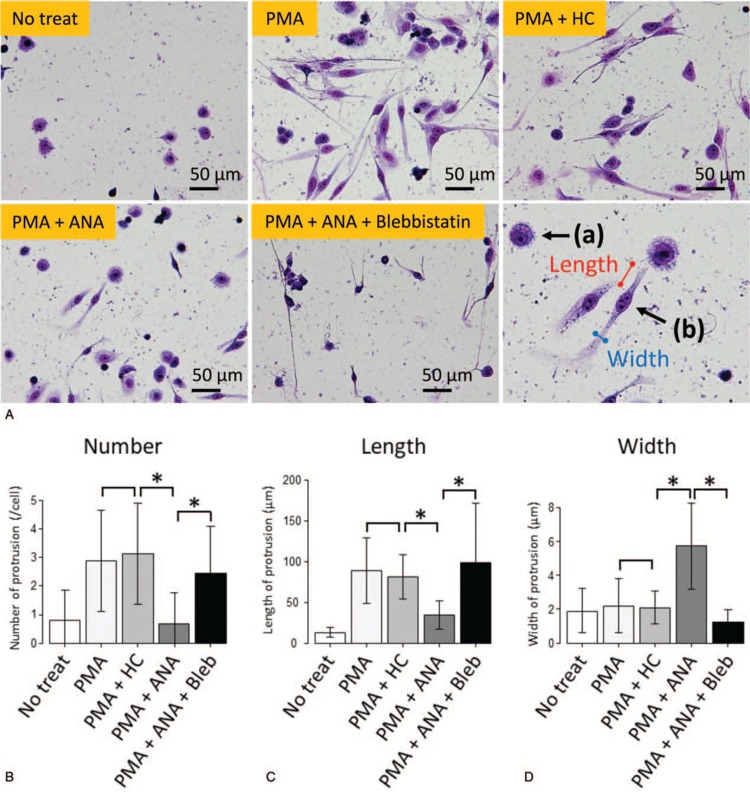
(A) May-Grünwald-Giemsa staining of MEG-01 cells. Upper left: non-treated MEG-01 cells, Upper middle: 10 nM PMA-treated MEG-01 cells, Upper right: 10 nM PMA and 100 μM HC-treated MEG-01 cells, Lower left: 10 nM PMA and 5 μM ANA-treated MEG-01 cells, Lower middle: 10 nM PMA, 5 μM ANA and 20 μM blebbistatin-treated MEG-01 cells, Lower right: example of count of morphological parameters. The addition of PMA led to extension of cytoplasmic protrusions (upper middle), the addition of ANA with PMA inhibited the extension of cytoplasmic protrusions (lower left), and the addition of HC with PMA did not cause any changes about cytoplasmic protrusions (upper right). The addition of blebbistatin with PMA + ANA led to extension of cytoplasmic protrusions (lower middle). The number of protrusions is 0 in cell (a) and the number of protrusions is 2 in cell (b). Red bar shows the length of protrusions and blue bar shows the width of protrusions (lower right). ANA = anagrelide; HC = hydroxycarbamide; PMA = phorbol-12-myristate-13-acetate. (B) The number of protrusions extending from MEG-01 cells was counted. The number of proplatelets was decreased when MEG-01 cells were treated with PMA + ANA than treated with PMA alone. The change was reversed when MEG-01 cells were treated with PMA + ANA + blebbistatin. (C) The length of cytoplasmic protrusions was measured. The cytoplasmic protrusions were shorter when MEG-01 cells were treated with PMA + ANA than treated with PMA alone. The change was reversed when MEG-01 cells were treated with PMA + ANA + blebbistatin. (D) The maximum shaft width of cytoplasmic protrusions was measured. The cytoplasmic protrusions were thicker when MEG-01 cells were treated with PMA+ANA than treated with PMA alone. The change was reversed when MEG-01 cells were treated with PMA + ANA + blebbistatin. For (B) to (D), 100 MEG-01 cells were analyzed in each sample. Results are means plus or minus SD from 3 independent experiments. ^∗^*P* < 0.001. ANA = anagrelide; HC = hydroxycarbamide; PMA = phorbol-12-myristate-13-acetate.

**Figure 4:**
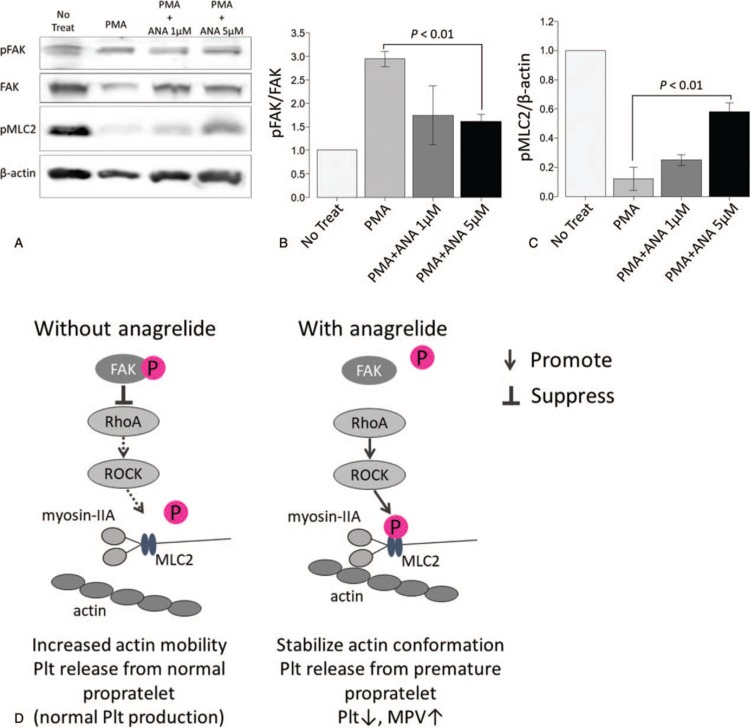
(A) Expression of pFAK, FAK, pMLC2, and β-actin in MEG-01 cells. Results are representative of 3 independent experiments for MEG-01 cells. (B) Relative quantification of the phosphorylation state of FAK. The phosphorylation state of FAK was quantified as a density ratio of protein bands, pFAK/FAK. Values of the samples were calibrated to that of a non-treated sample. Results are means plus or minus SD from 3 independent experiments. (C) Quantification of the phosphorylation state of MLC2. The phosphorylation state of MLC2 was quantified as a density ratio of protein bands, pMLC2/β-actin. Values of the samples were calibrated to that of a non-treated sample. Results are means plus or minus SD from 3 independent experiments. (D) Possible mechanism of the platelet-lowering effect of ANA. ANA suppresses phosphorylation of FAK and promotes phosphorylation of MLC2. As a result, proplatelet formation is interfered and platelet production is suppressed. ANA = anagrelide; FAK = focal adhesion kinase; pFAK = phosphorylated focal adhesion kinase; PMA = phorbol-12-myristate-13-acetate; pMLC2 = phosphorylated myosin light chain 2; Plt = platelet.

In order to determine how ANA affects the process of megakaryocyte differentiation, proplatelet formation, and PLP production, we performed an *in vitro* assay using MEG-01 cells. PMA-induced megakaryocytic differentiation was confirmed by detection of integrin αIIb and β3 expression using Western blotting (Fig. 2A). MEG-01 cells treated with PMA were stained with an anti-human CD61 (β3) antibody (Fig. 2B) for immunofluorescence staining. Following incubation of MEG-01 cells with PMA, the number of PLPs was decreased in the PMA + ANA treated MEG-01 cells (*P* < 0.01 vs PMA alone, Fig. 2B and C). PLP size was estimated by mean intensity of FS in PLPs. For PLP gating, a region was designated by the loading of human peripheral platelets following staining with a fluorescence-labeled anti-human CD41 (αIIb) antibody (data not shown). In the absence of PMA, slight staining of PLPs was detected in the region. The size of PLPs estimated by the intensity of FS using FCM was significantly increased in the PMA + ANA treated MEG-01 cells compared to the cells treated with PMA alone (*P* < 0.05, Fig. 2D and E). These results were consistent with the clinical findings in ET patients treated with ANA. The size of MEG-01 cells estimated by the intensity of FS using FCM was significantly decreased in the PMA + ANA treated MEG-01 cells compared to the cells treated with PMA alone (*P* < 0.01, Fig. 2F). We then focused on morphological changes of proplatelets induced by treatment with ANA. May-Grünwald-Giemsa staining of MEG-01 cells showed that the addition of PMA led to extension of cytoplasmic protrusions and that the addition of ANA with PMA inhibited the extension of cytoplasmic protrusions although the addition of HC with PMA had no influence on the morphology of cytoplasmic protrusions (Fig. 3A). Moreover, the addition of blebbistatin with PMA and ANA canceled the effect of ANA on cytoplasmic protrusions. To verify the morphological change, we compared morphological parameters including the number, length, and width of cytoplasmic protrusions as shown in Figure 3A (lower right). There was no difference in the number, length, and width of cytoplasmic prtrusions between MEG-01 cells treated with PMA alone and the cells treated with PMA + HC (*P* = 0.612, *P* = 0.496, *P* = 0.722, respectively, Fig. 3B–D). The cytoplasmic protrusions were shorter and thicker and the number of proplatelets was decreased when MEG-01 cells were treated with PMA+ANA (*P* < 0.001 vs PMA alone, Fig. 3B–D). The cytoplasmic protrusions were longer and thinner and the number of proplatelets was increased when MEG-01 cells were treated with PMA+ANA+blebbistatin (*P* < 0.001 vs PMA+ANA, Fig. 3B–D). The FAK-RhoA-ROCK-MLC2-myosin IIA pathway is an important pathway for the formation of proplatelets and production of platelets from megakaryocytes.^22–26^ We evaluated the effect of ANA on phosphorylation of FAK and MLC2. Western blotting analysis showed that ANA induced increased phosphorylation of MLC2 and reduced phosphorylation of FAK (PMA vs PMA+ANA 5 μM *P* < 0.01, Fig. 4A–C).

## Discussion

We found an interesting correlation between platelet reduction and MPV increase in patients treated with ANA. Although a previous study suggested that MPV was higher in ET patients treated with ANA than in those not treated with ANA,^27^ MPV change after ANA treatment was not analyzed. Our data clearly showed that successful platelet reduction by ANA correlated to increased MPV. This finding might be a clue for clarifying the mechanisms underlying the platelet-lowering effect of ANA. Cytoreduction by ANA is platelet-specific, and ANA does not suppress myeloid and erythroid lineages. In contrast, HC inhibits DNA synthesis and cytoreduction by HC is not lineage-specific, and thrombocyte reduction effect does not correlate to increase of MPV. Unanticipated anemia could be a major indicator of intolerance to HC in ET patients. ANA is known to inhibit PDEIII, but the levels of PDEIII inhibition and elevation of cAMP have no association with the platelet-lowering effect of ANA.^16,28^ ANA decreased platelet count without altering platelet function or thrombospondin content in case with ET.^29^ The change in size of platelets strongly suggested that ANA affected the final process of platelet production. Thus, we hypothesized that ANA modifies the process by which platelets are released from proplatelets. Since the platelet-lowering effect of ANA is a human-specific phenomenon, we verified the finding using the human megakaryocytic cell line MEG-01, which can be differentiated to megakaryocytes by PMA treatment. ANA reduced PLP production and the size of PLPs was confirmed to be increased, recapitulating clinical findings. The morphological change of cells after ANA treatment was striking. MEG-01 cells form propletlets and release PLPs after megakaryocytic differentiation induced by PMA. ANA suppressed proplatelet formation, resulting in decreased number and shortened length of proplatelet. Short and thick protrusions may contribute to the decreased number and increased size of PLPs. A previous study showed that ANA suppressed transcription factors responsible for megakaryocytic differentiation such as *FLI-1, FOG-1,* and *GATA-1*^13,14^ that resulted in smaller megakaryocytes.^11^ Platelet size change could not be explained just by suppression of megakaryopoiesis. Besides blocking megakaryopoiesis, our study showed that ANA exerted a direct inhibitory effect on thrombopoiesis.

Proplatelet formation process depends on actin and microtubule dynamics. While microtubules are used to propel proplatelet elongation, an actin-dependent reaction is used to bifurcate the proplatelet shaft, thereby increasing the number of proplatelet tips available to participate in platelet formation.^30^ The FAK-RhoA-ROCK-MLC2-myosin IIA pathway plays an important role controlling actin dynamics in the process of proplatelet formation.^22,23,25^ An association between decreased platelet count and increased platelet size was reported in congenital macrothrombocytopenia, in which the FAK-RhoA-ROCK-MLC2-myosin IIA pathway was disregulated.^23–26^ Heterozygous mutation at the membrane proximal region of *ITGA2B* or *ITGB3* causes constitutional partial activation of the integrin αIIbβ3 complex and premature release of platelets, resulting in production of giant platelets. We show a possible mechanism of the platelet-lowering effect of ANA in Figure 4D. Forced expression of the mutated integrin resulted in abnormal cytoplasmic protrusions similar to those in ANA-treated MEG-01 cells.^25^ In megakaryocytes, phosphorylation of FAK negatively controls the RhoA-ROCK-MLC2-myosin IIA pathway and leads to proplatelet formation and platelet production.^22–25^ In this study, we revealed decreased phosphorylation of FAK and increased phosphorylation of MLC2 in MEG-01 cells treated with ANA. These findings indicated that ANA modulated the FAK-RhoA-ROCK-MLC2-myosine IIA pathway, leading to abnormal proplatelet formation that resulted in a decrease of platelet count and increase of MPV. A decreased level of phosphorylated FAK promotes the RhoA-ROCK-MLC2-myosinIIA pathway and phosphorylated MLC2 stabilizes actin conformation, resulting in poor mobility of the cell surface and reduced platelet production. Some studies in patients with macrothrombocytopenia showed that insufficient activation of integrin αIIbβ3 and platelet production from immature proplatelets led to the production of larger platelets.^25,26^ Based on the similar change in the number and size of platelets in macrothrombocytopenia, we hypothesizes that ANA targets the FAK-RhoA-ROCK-MLC2-myosine IIA pathway as well. Based on ANA's rapid onset of action, besides blocking megakaryopoiesis, repression of proplatelet formation might be the main mechanism underlying its platelet-lowering effect. Other possibility is the action mediated by microtubules. Direct inhibitor of microtubule, such as colchicine, prevent proplatelet formation. Change of microtubule bundling or elastic bending could potentially affect platelet size.^31^ We found morphological change induced by ANA was reversed by blebbistatin, a specific myosin II inhibitor. So, we speculated that RhoA-ROCK-MLC2-myosine IIA-Actin pathway were responsible for clinical effect of ANA treatment. Our study revealed that ANA affects the process of proplatelet formation through modification of FAK-RhoA-ROCK-MLC2-myosine IIA pathway.

## Conclusion

ANA reduces platelet counts and increases platelet size by suppressing proplatelet formation through modulation of the FAK-RhoA-ROCK-MLC2-myosin IIA pathway. Morphological observation suggests that ANA induces premature release of platelets, resulting in increased platelet size. Further work on the mechanisms underlying the thrombocytopenic effects of ANA may contribute to elucidation of the pathways regulating platelet biogenesis.

## Supplementary Material

Supplemental Digital Content

## Supplementary Material

Supplemental Digital Content

## References

[1] Barbui T Vannucchi AM Buxhofer-Ausch V Practice-relevant revision of IPSET-thrombosis based on 1019 patients with WHO-defined essential thrombocythemia. *Blood Cancer J.* 2015;5:e369. 26617062 10.1038/bcj.2015.94 PMC4670947

[2] Barbui T Finazzi G Carobbio A Development and validation of an International Prognostic Score of thrombosis in World Health Organization-essential thrombocythemia (IPSET-thrombosis). *Blood.* 2012;120:5128–5133. quiz 5252. 23033268 10.1182/blood-2012-07-444067

[3] Ruggeri M Finazzi G Tosetto A No treatment for low-risk thrombocythaemia: results from a prospective study. *Br J Haematol.* 1998;103:772–777. 9858229 10.1046/j.1365-2141.1998.01021.x

[4] Barbui T Tefferi A Vannucchi AM Philadelphia chromosome-negative classical myeloproliferative neoplasms: revised management recommendations from European LeukemiaNet. *Leukemia.* 2018;32:1057–1069. 29515238 10.1038/s41375-018-0077-1 PMC5986069

[5] Mesa RA Jamieson C Bhatia R NCCN Guidelines Insights: Myeloproliferative Neoplasms, Version 2.2018. *J Natl Compr Canc Netw.* 2017;15:1193–1207. 28982745 10.6004/jnccn.2017.0157

[6] Dombi P Illes A Demeter J Anagrelide reduces thrombotic risk in essential thrombocythaemia vs. hydroxyurea plus aspirin. *Eur J Haematol.* 2017;98:106–111. 27557754 10.1111/ejh.12806

[7] Gisslinger H Gotic M Holowiecki J Anagrelide compared with hydroxyurea in WHO-classified essential thrombocythemia: the ANAHYDRET Study, a randomized controlled trial. *Blood.* 2013;121:1720–1728. 23315161 10.1182/blood-2012-07-443770 PMC3591796

[8] Harrison CN Campbell PJ Buck G Hydroxyurea compared with anagrelide in high-risk essential thrombocythemia. *N Engl J Med.* 2005;353:33–45. 16000354 10.1056/NEJMoa043800

[9] Fleming JS Buyniski JP A potent new inhibitor of platelet aggregation and experimental thrombosis, anagrelide (BL-4162A). *Thromb Res.* 1979;15:373–388. 115106 10.1016/0049-3848(79)90145-2

[10] Abe Andes W Noveck RJ Fleming JS Inhibition of platelet production induced by an antiplatelet drug, anagrelide, in normal volunteers. *Thromb Haemost.* 1984;52:325–328. 6531755

[11] Mazur EM Rosmarin AG Sohl PA Analysis of the mechanism of anagrelide-induced thrombocytopenia in humans. *Blood.* 1992;79:1931–1937. 1562721

[12] Solberg LA Jr Tefferi A Oles KJ The effects of anagrelide on human megakaryocytopoiesis. *Br J Haematol.* 1997;99:174–180. 9359521 10.1046/j.1365-2141.1997.3503164.x

[13] Ahluwalia M Donovan H Singh N Anagrelide represses GATA-1 and FOG-1 expression without interfering with thrombopoietin receptor signal transduction. *J Thromb Haemost.* 2010;8:2252–2261. 20586925 10.1111/j.1538-7836.2010.03970.x

[14] Sakurai K Fujiwara T Hasegawa S Inhibition of human primary megakaryocyte differentiation by anagrelide: a gene expression profiling analysis. *Int J Hematol.* 2016;104:190–199. 27084257 10.1007/s12185-016-2006-2

[15] Thon JN Italiano JE Platelet formation. *Semin Hematol.* 2010;47:220–226. 20620432 10.1053/j.seminhematol.2010.03.005 PMC2904625

[16] Espasandin YR Glembotsky AC Grodzielski M Anagrelide platelet-lowering effect is due to inhibition of both megakaryocyte maturation and proplatelet formation: insight into potential mechanisms. *J Thromb Haemost.* 2015;13:631–642. 25604267 10.1111/jth.12850

[17] Isakari Y Sogo S Ishida T Gene expression analysis during platelet-like particle production in phorbol myristate acetate-treated MEG-01 cells. *Biol Pharm Bull.* 2009;32:354–358. 19252277 10.1248/bpb.32.354

[18] Takeuchi K Satoh M Kuno H Platelet-like particle formation in the human megakaryoblastic leukaemia cell lines, MEG-01 and MEG-01s. *Br J Haematol.* 1998;100:436–444. 9488640 10.1046/j.1365-2141.1998.00576.x

[19] Takeuchi K Ogura M Saito H Production of platelet-like particles by a human megakaryoblastic leukemia cell line (MEG-01). *Exp Cell Res.* 1991;193:223–226. 1995298 10.1016/0014-4827(91)90560-h

[20] Lev PR Grodzielski M Goette NP Impaired proplatelet formation in immune thrombocytopenia: a novel mechanism contributing to decreased platelet count. *Br J Haematol.* 2014;165:854–864. 24673454 10.1111/bjh.12832

[21] Kanda Y Investigation of the freely available easy-to-use software ’EZR’ for medical statistics. *Bone Marrow Transplant.* 2013;48:452–458. 23208313 10.1038/bmt.2012.244 PMC3590441

[22] Chang Y Aurade F Larbret F Proplatelet formation is regulated by the Rho/ROCK pathway. *Blood.* 2007;109:4229–4236. 17244674 10.1182/blood-2006-04-020024

[23] Chen Z Naveiras O Balduini A The May-Hegglin anomaly gene MYH9 is a negative regulator of platelet biogenesis modulated by the Rho-ROCK pathway. *Blood.* 2007;110:171–179. 17392504 10.1182/blood-2007-02-071589 PMC1896110

[24] Kobayashi Y Matsui H Kanai A Identification of the integrin beta3 L718P mutation in a pedigree with autosomal dominant thrombocytopenia with anisocytosis. *Br J Haematol.* 2013;160:521–529. 23253071 10.1111/bjh.12160

[25] Miyashita N Onozawa M Hayasaka K A novel heterozygous ITGB3 p.T720del inducing spontaneous activation of integrin alphaIIbbeta3 in autosomal dominant macrothrombocytopenia with aggregation dysfunction. *Ann Hematol.* 2018;97:629–640. 29380037 10.1007/s00277-017-3214-4

[26] Kunishima S Kashiwagi H Otsu M Heterozygous ITGA2B R995W mutation inducing constitutive activation of the alphaIIbbeta3 receptor affects proplatelet formation and causes congenital macrothrombocytopenia. *Blood.* 2011;117:5479–5484. 21454453 10.1182/blood-2010-12-323691

[27] Kissova J Bulikova A Ovesna P Increased mean platelet volume and immature platelet fraction as potential predictors of thrombotic complications in BCR/ABL-negative myeloproliferative neoplasms. *Int J Hematol.* 2014;100:429–436. 25227185 10.1007/s12185-014-1673-0

[28] Wang G Franklin R Hong Y Comparison of the biological activities of anagrelide and its major metabolites in haematopoietic cell cultures. *Br J Pharmacol.* 2005;146:324–332. 16041400 10.1038/sj.bjp.0706341 PMC1576287

[29] Bellucci S Legrand C Boval B Studies of platelet volume, chemistry and function in patients with essential thrombocythaemia treated with Anagrelide. *Br J Haematol.* 1999;104:886–892. 10192455 10.1046/j.1365-2141.1999.01234.x

[30] Italiano JE Jr Patel-Hett S Hartwig JH Mechanics of proplatelet elaboration. *J Thromb Haemost.* 2007;5 Suppl 1:18–23. 17635704 10.1111/j.1538-7836.2007.02487.x

[31] Thon JN Macleod H Begonja AJ Microtubule and cortical forces determine platelet size during vascular platelet production. *Nat Commun.* 2012;3:852. 22617292 10.1038/ncomms1838

