# Supplementary material for: Anagrelide Modulates Proplatelet Formation Resulting in Decreased Number and Increased Size of Platelets
Source: Hemasphere. 2019 Jun 18;3(4):e268. doi: 10.1097/HS9.0000000000000268 (PMC6745917; doi:10.1097/HS9.0000000000000268)
Supplement: Supplemental Digital Content [file hs9-3-e268-s002.pdf]

Supplemental Figure 1

(A)

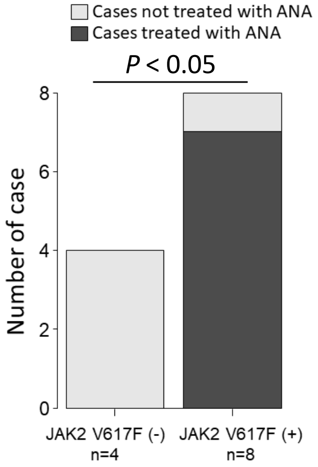

(B)

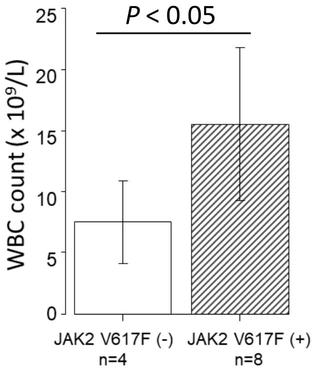

(C)

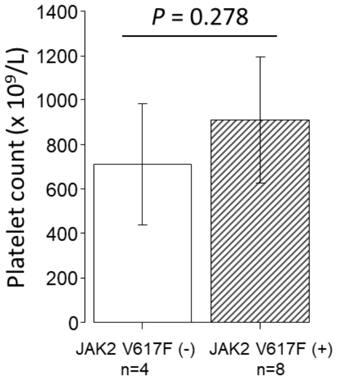

(D)

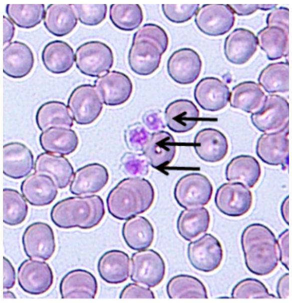

(A) The percentage of administration of ANA. Patients were stratified into a *JAK2* V617F-positive group and a *JAK2* V617F-negative group. ANA, anagrelide.

(B) WBC count at diagnosis of ET. Patients were stratified into a *JAK2* V617F-positive group and a *JAK2* V617F-negative group. WBC, white blood cell.

(C) Platelet count at diagnosis of ET. Patients were stratified into a *JAK2* V617F-positive group and a *JAK2* V617F-negative group.

(D) Peripheral blood smear of a patient after initiation of ANA treatment. The arrows indicate giant platelets.
